# Brain-imaging during an isometric leg extension task at graded intensities

**DOI:** 10.3389/fphys.2013.00296

**Published:** 2013-10-18

**Authors:** Vera Abeln, Alexandra Harig, Axel Knicker, Tobias Vogt, Stefan Schneider

**Affiliations:** ^1^Institute of Movement and Neurosciences, German Sport University Cologne, Cologne, Germany; ^2^Faculty of Science, Health, Education and Engineering, University of the Sunshine Coast, Maroochydore, QLD, Australia

**Keywords:** electroencephalography, sLORETA, electromyography, high-intensity exercise, whole-body exercise, cortical current density, motor cortex, sensorimotor cortex

## Abstract

Imaging the brain during complex and intensive movements is challenging due to the susceptibility of brain-imaging methods for motion and myogenic artifacts. A few studies measured brain activity during either single-joint or low-intensity exercises; however, the cortical activation state during larger movements with increases up to maximal intensity has barely been investigated so far. Eleven right-handed volunteers (22–45 years in age) performed isometric leg extensions with their right leg at 20, 40, 60, 80, and 100% of their maximal voluntary contraction. Contractions were hold for 20 s respectively. Electroencephalographic (EEG) and electromyographic (EMG) activity was recorded. Standardized low-resolution brain electromagnetic tomography (sLORETA) was used to localize the cortical current density within the premotor (PMC), primary motor (M1), primary somatosensory (S1) and somatosensory association cortex (SAC). ANOVA was used for repeated measures for comparison of intensities and between the left and right hemispheres. The quality of the EEG signal was satisfying up to 80% intensity. At 100% half of the participants were not able to keep their neck and face muscles relaxed, leading to myogenic artifacts. Higher contralateral vs. ipsilateral hemispheric activity was found for the S1, SAC and, PMC. M1 possessed higher ipsilateral activity. The highest activity was localized in the M1, followed by S1, PMC, and SAC. EMG activity and cortical current density within the M1 increased with exercise intensity. EEG recordings during bigger movements up to submaximal intensity (80%) are possible, but maximal intensities are still hard to investigate when subjects contracted their neck and face muscles at the same time. Isometric contractions mainly involve the M1, whereas the S1, PMC, and SAC seem not to be involved in the force output. Limitations and recommendations for future studies are discussed.

## Introduction

For understanding how the brain initiates and organizes exercise, it is essential to monitor the brain at work. Imaging the brain during complex and intensive movements is challenging due to the susceptibility of current brain-imaging methods for motion and myogenic artifacts, such as functional magnet resonance imaging (fMRI) or positron emission tomography (PET).

While exercise-induced changes of brain-cortical function from pre- to post-exercise have extensively been accomplished (Crabbe and Dishman, 2004), literature investigating the brain during exercise is mainly restricted to studies measuring brain activity during either single-joint and/or low-intensity exercises. For instance, Christensen et al. (2000) localized increased activity within the primary sensory (S1), primary motor (M1), and supplementary motor cortex during moderate pedaling exercise using PET. Different cortical activation patterns were observed comparing elbow and knee flexion/extension by fMRI (Kashima et al., 2013). Another fMRI study revealed increased activity within the left M1 and the dorsolateral premotor cortex (PMC) during repeated isometric knee extension and ankle plantar- and dorsiflexion (Newton et al., 2008). Higher supplementary motor and dorsolateral PMC activity was recorded during knee extension in comparison to dorsiflexion. That different kinds of contraction involve different cortical activation patterns was detected by Gwin and Ferris (2012). While isometric knee and ankle flexion/extension resulted in spectral modulations at joint torque onset and offset only, sustained desynchronization was found during isotonic contractions throughout the trial.

However, the cortical activation state during whole-body and high-intensity movements, up to maximal intensity, has barely been investigated so far. For this purpose, either functional near-infrared spectroscopy (fNIRS) or electroencephalography (EEG) has been applied promising to be less susceptible for motion artifacts. fNIRS has been proven allow monitoring of brain activity during resistance exercise and to reveal comparable results to fMRI (Pereira et al., 2007), however, the correlation and interfering factors between cerebral oxygenation and electrocortical activity are still unclear (Devor et al., 2003). Using EEG, a number of studies discussed changes within specific frequency ranges during bicycling exercise up to maximal intensities (Kubitz and Mott, 1996; Nybo and Nielsen, 2001; Bailey et al., 2008). As findings for the frequency bands are controversial, the meaning of changes within these bands has not been clarified (Crabbe and Dishman, 2004).

A previous study was able to show increasing cortical current density (CCD) within the M1 with increasing bicycling exercise intensity, proving that EEG recordings and localization during maximal intensity exercise is possible (Brummer et al., 2011). Lately, Mekjavic et al. (2005); Schneider et al. (2013) were even able to match increasing EEG and electromyography (EMG) activity with elevated bicycling intensity. Similar brain-imaging attempts and correlations between EEG and EMG activity have not been accomplished for strength exercise so far.

Not only for our basic understanding of brain function, but also for the exploration of movement disorders up to the improvement of performance, it is worth to study the brain during high intensity exercises and to clarify which brain regions are instrumental in executing and controlling an isometric contraction. Moreover, it is intended to test whether it is feasible to record and localize such changes using EEG as a simple and economic alternative to magnet-resonance-imaging. The aim of this study was to investigate motor and sensorimotor brain areas during unilateral isometric leg extensions at 20% up to 100% intensity. Based on the literature and the somatotopic organization of the motor and sensorimotor cortex, it was hypothesized to find increased EMG and contralateral EEG activity with exercise intensity within M1 and higher contralateral activity within S1.

## Materials and methods

### Study design

The local ethic committee approved the study. Eleven volunteers (22–45 years, seven males) were informed about the aim of the study and afterwards signed an informed consent form. All of them were right-handed according to the Edinburgh Handedness Inventory (Oldfield, 1971). All participants declared to be healthy and to never have experienced any neurological or sensorimotor dysfunctions. Tests were performed in an air-conditioned and quiet laboratory. Beforehand, participants were familiarized with the test conditions and devices.

A 32- channel flexible EEG cap (ActiCap, BrainProducts GmbH, Munich, Germany) was placed on participants heads based on the 10–20 system of Jasper (1958). The cap contained the electrodes FP1, FP2, F7, F3, Fz, F4, F8, FC5, FC1, FC2, FC6, T7, C3, Cz, C4, T8, TP9, CP5, CP1, CP2, CP6, TP10, P7, P3, Pz, P4, P8, PO9, O1, Oz, O2 und PO10 and was fixed by a chinstrap to avoid electrode shifts. Electrodes were filled with gel (EasyCap SuperVisc, EasyCap GmbH, Herrsching, Germany) and handled by a blunt-tip needle until impedance values below 5 kilo ohm were reached. Two EMG electrodes were attached above the right Musclus (M.) vastus medialis and M. vastus lateralis on the shaved and cleaned skin of participants' thighs. EMG was integrated into the EEG configuration in order to record electromyographic activity in parallel to EEG activity. Participants were seated on an isokinetic dynamometer (IsoMed 2000, D&R GmbH, Hamburg, Germany). The cushion of the lever arm of the dynamometer was fixed via hook-and-loop tape to the lower leg. Additionally, two tapes fixed the thigh and the hip to the seat. The rotary axis of the lever arm was on level of the knee joint.

For warm-up, 20 dynamic trials with light to moderate loads were executed at first. The intended task for the participants was to perform isometric leg extensions with their right leg at 20, 40, 60, 80, and 100% of their maximal voluntary contraction. Participants absolved the order of intensities randomly. For the intended tests, the angle of the lever arm was stationary at 110° (interior knee angle). Three consecutive isometric maximal trials were performed for evaluation of the individual maximal voluntary capacity. Trials were repeated with a one-min rest in-between. Afterwards, a five-min rest was given. The mean out of the three trials was set as 100% and the corresponding 20 to 80% intensities were calculated. A target line on the screen in front of the participants provided the intended intensity, which participants were asked to follow with their produced strength line.

Once the target line was reached, EEG and EMG recordings were started with 500 Hz recording frequency for 20 s while participants held the contraction on the target line. Recording was performed using the Brain Vision Recorder (Brain Products GmbH, Munich, Germany). After 20 s, recordings were stopped and then subjects released contraction. Following each trial, participants rested for three min before performing the next intensity. Subjects were asked to keep their upper body and especially their shoulder, neck and face muscles as relaxed as possible to avoid co-contractions and myogenic artifacts within the EEG.

### Analysis

Data preparations and analyses were performed using Brain-Vision-Analyzer 2.0 (BrainProducts GmbH, Munich, Germany). The 20 s EEG measurements were first filtered by Butterworth Zero Phase Filter (low-pass: 3.5 Hz; high-pass: 70 Hz; 48 dB/oct; Notch filter: 50 Hz). Single noisy electrodes were replaced via topographic interpolation. Using independent component analysis, eye-movements were subtracted. Data was segmented into one-s equal sized segments with 10% allowed overlap. Artifacts were removed using first, the automatic artifact rejection (gradient criteria: 50 μV/ms, difference criteria: 200 μV, minimal allowed amplitude: −200 μV, maximal allowed amplitude: 200 μV; low activity criteria: lowest allowed activity in intervals 0.5 μV, interval length: 200 ms, marked bad events 200 ms before and after), followed by a manual artifact rejection scanning the data visually. Data was finally baseline corrected and localized within the regions of interest by the integrated standardized low-resolution brain electromagnetic tomography (sLORETA) function of the Brain Vision Analyzer 2.0 [for more information regarding sLORETA please see the work of Pascual-Marqui and colleagues (Pascual-Marqui et al., 1999, 2002; Pascual-Marqui, 2002)]. The CCD within a 9 mm sphere around the regions of interest was exported as rectified raw sum of activity values (μV^2^/mm^4^) averaged over the remaining segments. The regions of interest were:

- premotor cortex (PMC), coordinates −10, +10/10/60
- primary motor cortex (M1), coordinates −10, +10/0/60
- primary somatosensory cortex (S1), coordinates −10, +10/−20/60 and
- somatosensory association cortex (SAC), coordinates −10, +10/−40/60.

The given coordinates represent the *x/y/z*- axis (*x*: width, *y*: length, *z*: height) according to the Montreal Neurological Institute (MNI) and are based on previous work about knee extension (Hanakawa et al., 1999; Christensen et al., 2000; Gerardin et al., 2003; Gwin and Ferris, 2012) and the brain atlas of Talairach and Tournoux (1988). In addition, identifying the region of the highest activity for each individual respectively checked the correct localization of the motor cortex.

EMG data was filtered by Butterworth Zero Phase filter (low-pass: 20 Hz, high-pass: 249 Hz, 48dB/oct; Notch filter: 50 Hz). Data was segmented into 4 s equal sized segments, baseline corrected, rectified and averaged afterwards. Activity was exported as mean activity (μV).

### Statistics

ANOVA for repeated measures was used for comparison of CCD between the left and right hemisphere (factor: HEMI) as well as between intensities (factor: INT). EMG activity was also compared between intensities by ANOVA for repeated measures. Threshold of significance was *p* < 0.05 (^*^), *p* < 0.01 (^**^), *p* < 0.001 (^***^). Fisher LSD *post-hoc* test was used in case of significance.

## Results

### EEG

The quality of the EEG signal was satisfying up to 80% intensity. At 100%, more than half of the participants were not able to keep their neck and face muscles relaxed leading to motion and myogenic artifacts. Therefore, 100% intensity was excluded from the analysis.

**Figure 1:**
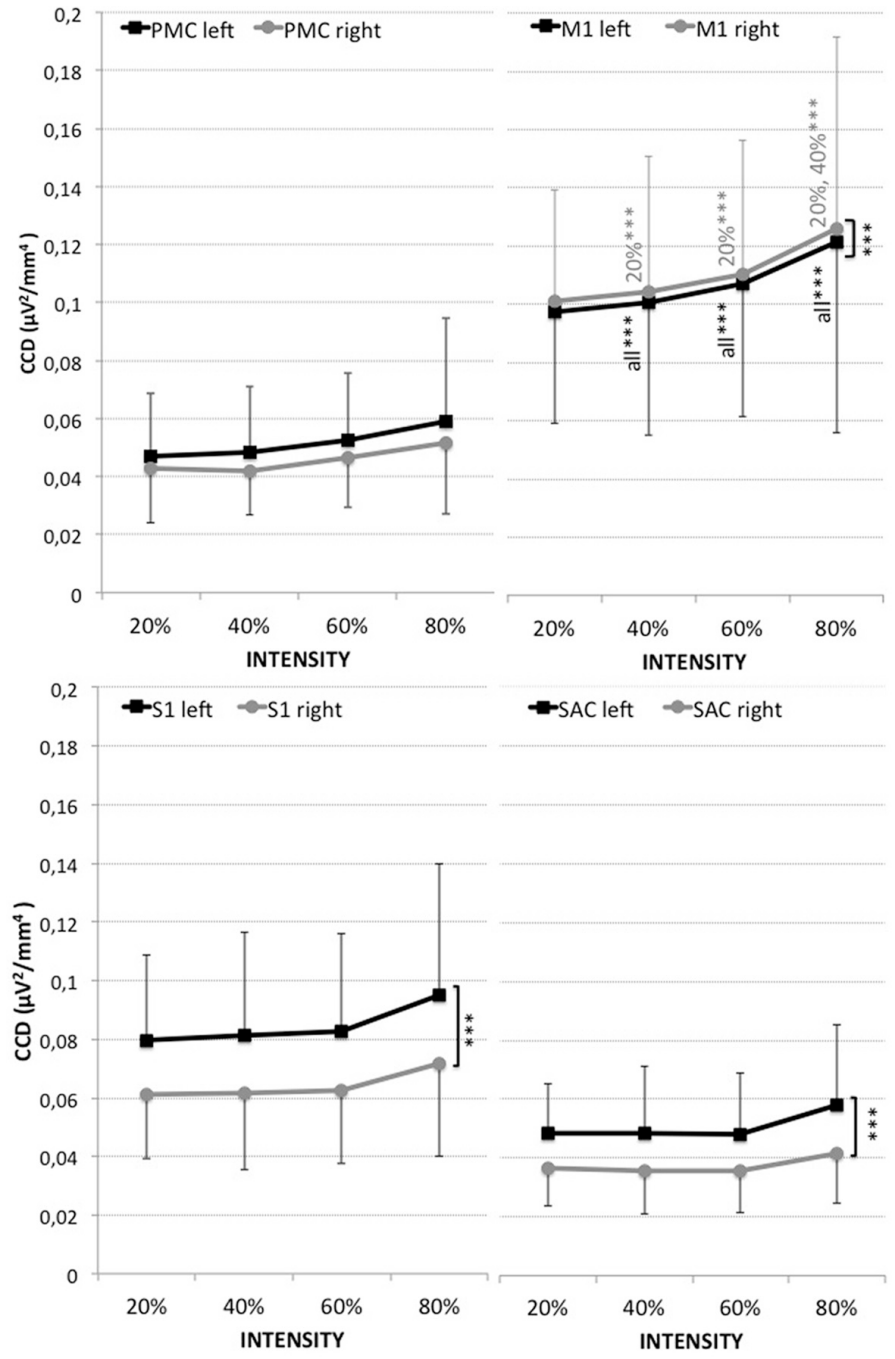
**Cortical current density (CCD, μV^2^/mm^4^) of the four regions of interest (PMC, M1, S1, SAC) within the left (black) and right (gray) hemisphere for measurements at 20, 40, 60, and 80% of the maximal voluntary contraction during leg extension.** Results are presented as mean ± standard deviation. Significant differences are indicated by ^***^p < 0.001. Corresponding results of ANOVA are presented in Table 1.

The highest activity was localized in the right hemisphere directly followed by the left hemisphere of the M1, followed by the left then right hemispheres of the S1, the left hemisphere of the PMC, the left hemisphere of the SAC, and finally the right hemisphere of the PMC and SAC (see Figure 1).

A significant effect for hemisphere (HEMI) was revealed for the M1, S1, and SAC (see Figure 1 and Table 1). Higher left (contralateral) vs. right (ipsilateral) hemispheric activity was found within the S1 and SAC. Higher activity within the right hemisphere (ipsilateral) than left hemisphere was revealed for the M1.

**Table 1:** **Results of ANOVA for repeated measures comparing the differences of the EMG (M. vastus medialis and lateralis) and EEG activity of the regions of interest between intensities (INT: rest, 20, 40, 60, 80%), and for EEG also between hemispheres (HEMI: left, right)**.

	**Effect**	***F***	***p***	
M. vastus medialis	INT	28.409	<0.001	^***^
M. vastus lateralis	INT	9.524	<0.001	^***^
PMC	INT	2.560	0.076	
	HEMI	3.464	0.096	
M1	INT	3.110	0.043	^*^
	HEMI	45.632	<0.001	^***^
S1	INT	2.733	0.063	
	HEMI	45.010	<0.001	^***^
SAC	INT	1.339	0.282	
	HEMI	38.130	<0.001	^***^

Given are F- and p-values.

Significant effects are indicated by

* p <0.05 or

*** p < 0.001.

CCD increased with leg extension intensity within the M1. No significant effect for intensity was found for the S1, PMC, and SAC.

### EMG

**Figure 2:**
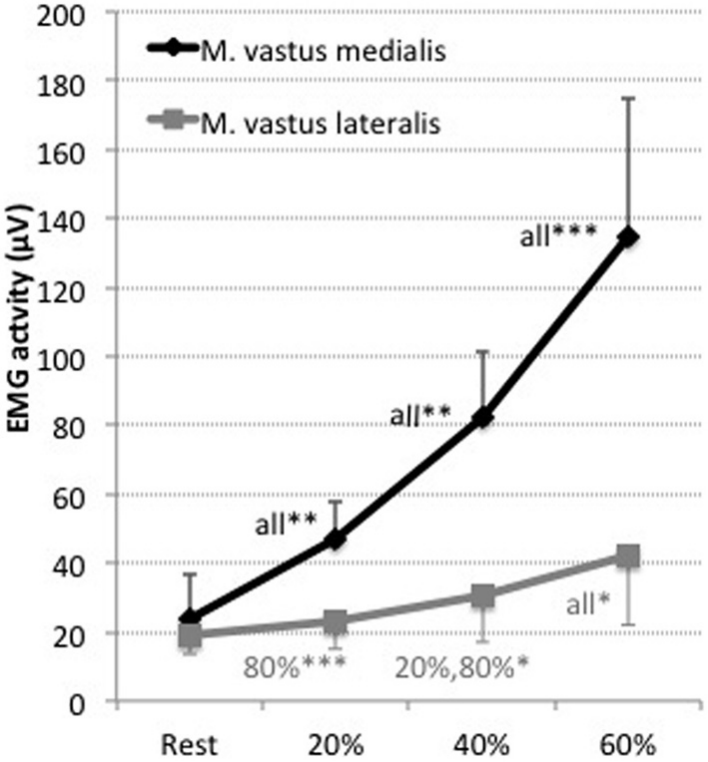
**EMG activity (μV) of the Musculus vastus medialis (black) and Musculus vastus lateralis (gray) for the measurements at 20, 40, 60, and 80% of the maximal voluntary contraction during leg extension.** Shown are the mean values and the standard deviations (bars). Significant differences are indicated by ^*^p < 0.05, ^**^p < 0.01, ^***^p < 0.001 plus the corresponding measurement it is different to. Results are presented as mean ± standard deviation. Corresponding results of ANOVA are presented in Table 1.

Electromyographic activity increased from 20% within both, M. vastus medialis and lateralis, with leg extension intensity (see Figure 2 and Table 1). Higher activity was observed within the M. vastus medialis, for which measurements at 40% (60%: *p* < 0.01; for 80%: *p* < 0.001), 60% (20, 80%: *p* < 0.001) and 80% (for all *p* < 0.001) significantly differed compared to all remaining measurements respectively. For M. vastus lateralis, increases were found for 40% compared to 80% (*p* < 0.001), for 60% compared to 20% (*p* < 0.05) and 80% (*p* < 0.05), as well as for 80% compared to all other measurements (20, 40%: *p* < 0.001).

## Discussion

This study aimed to investigate EMG activity within the involved muscles and EEG activity within motor and somatosensory brain regions during an isometric unilateral leg extension task with graded, up to maximal intensity. Results indicate that EEG recording during strength exercise up to submaximal intensity (80%) is possible, but maximal intensities are still hard to investigate when subjects co-contract their neck and face muscles. Nevertheless, the method of EEG combined with sLORETA extends the opportunities for brain-imaging during intensive exercises, which are restricted using current brain-imaging techniques such as fMRI or PET due to the necessity of holding the head steady. Additional EMG electrodes above the corresponding muscles around the head might help for future studies to subtract electromyographic from electrocortical activity.

EMG results demonstrate that higher isometric force output is going along with higher electromyographic activity. Correspondingly, elevations of M1 activity with leg extension intensity were found. Thus, the M1 seems to be involved in the force output as shown before (Christensen et al., 2000; Newton et al., 2008; Brummer et al., 2011; Schneider et al., 2013).

Ipsilateral activation within the M1 during knee extensions has been observed before (Kashima et al., 2013), however, that ipsilateral activation was higher than contralateral activation was not expected. As it cannot be distinguished between exhibitory or inhibitory activation, the higher ipsilateral activation of the M1 might occur due to the inhibition of the non-involved (left) leg. Furthermore, it is known that some nerve fibers of the pyramidal tract do not decussate to the contralateral side as well as that extrapyramidal fibers do not decussate, which are mainly in charge for regulation of bulky movements of the trunk and proximal extremities. Thus, the co-activation of the trunk muscles as well as activation of non-crossing pyramidal nerve fibers might account for the high ipsilateral activity. It might also be speculated—as no EMG measurements of the left leg have been performed—that the left leg was contracted, too, as reported before (Mayston et al., 1999; Beaule et al., 2012). Additionally, imprecision of localization could result out of the application of standardized brain maps and coordinates for the regions of interest as well as the striking distance of the leg area on the homunculus to the medial longitudinal fissure. Although consulted brain models for localization have been validated in numerous studies (Mulert et al., 2004; Bai et al., 2007), laying the EEG data on top of individual brain scans might impede potential mapping errors and should be regarded in subsequent studies.

The S1 shows a distinct higher contralateral activation as expected based on its somatotopic organization and its crossing nerve fibers to the contralateral side. The missing effect of intensity supports the assumption (Christensen et al., 2000; Brummer et al., 2011) that the S1 is not involved in force output.

The PMC did not show significant elevations of activity with exercise intensity. At joint torque onset and offset, Gwin and Ferris found an event related desynchronization (Gwin and Ferris, 2012) for isometric knee extensions, but a sustained desynchronization during isotonic contractions. Within this study, joint torque onset and offset was not included. The PMC is known to process motor planning but not the execution, thus it was not expected to increase activation with intensity.

The SAC is assumed to process the integration of visual and proprioceptive information of the participants trying to hold the produced force line on the intended target line with visual feedback online. This assumption is based on previous observations (Hanakawa et al., 1999; Sugawara et al., 2013). Higher activation of the contralateral hemisphere is probably caused by the nearness to the somatotopic organized S1.

Finally, it should be pointed out—although differences of activation between ROIs and hemispheres could be identified—that the resolution of sLORETA based on a 32-channel EEG system is limited. EEG caps with higher electrode density are recommended for future investigations.

In conclusion, this preliminary study suggests that EEG combined with sLORETA allows localizing electrocortical activity during isometric strength tasks up to submaximal intensity (80%) and paves the way for further brain-imaging studies of whole-body and high-intensity exercise. The findings suggest that unilateral isometric knee extensions with graded intensities require significant higher brain cortical activity within the M1, but not S1, PMC, and SAC. Accompanying measures of electromyographic activity of the non-used leg, neck, and face muscles, and of individual brain anatomy, as well as a higher density of electrodes, are recommended for future studies.

### Conflict of interest statement

The authors declare that the research was conducted in the absence of any commercial or financial relationships that could be construed as a potential conflict of interest.
